# Increased Understanding of Stem Cell Behavior in Neurodegenerative and Neuromuscular Disorders by Use of Noninvasive Cell Imaging

**DOI:** 10.1155/2016/6235687

**Published:** 2016-02-22

**Authors:** Bryan Holvoet, Liesbeth De Waele, Mattia Quattrocelli, Olivier Gheysens, Maurillio Sampaolesi, Catherine M. Verfaillie, Christophe M. Deroose

**Affiliations:** ^1^Nuclear Medicine and Molecular Imaging, Department of Imaging and Pathology, KU Leuven, 3000 Leuven, Belgium; ^2^Department of Development and Regeneration, Kulak Kortrijk, Department of Paediatric Neurology, University Hospitals Leuven, 3000 Leuven, Belgium; ^3^Translational Cardiomyology Lab, Department of Development and Regeneration, KU Leuven, 3000 Leuven, Belgium; ^4^Stem Cell Institute Leuven, Department of Development and Regeneration, KU Leuven, 3000 Leuven, Belgium; ^5^UZ Leuven, Division of Nuclear Medicine, Campus Gasthuisberg, Herestraat 49, 3000 Leuven, Belgium

## Abstract

Numerous neurodegenerative and neuromuscular disorders are associated with cell-specific depletion in the human body. This imbalance in tissue homeostasis is in healthy individuals repaired by the presence of endogenous stem cells that can replace the lost cell type. However, in most disorders, a genetic origin or limited presence or exhaustion of stem cells impairs correct cell replacement. During the last 30 years, methods to readily isolate and expand stem cells have been developed and this resulted in a major change in the regenerative medicine field as it generates sufficient amount of cells for human transplantation applications. Furthermore, stem cells have been shown to release cytokines with beneficial effects for several diseases. At present however, clinical stem cell transplantations studies are struggling to demonstrate clinical efficacy despite promising preclinical results. Therefore, to allow stem cell therapy to achieve its full potential, more insight in their* in vivo* behavior has to be achieved. Different methods to noninvasively monitor these cells have been developed and are discussed. In some cases, stem cell monitoring even reached the clinical setting. We anticipate that by further exploring these imaging possibilities and unraveling their* in vivo* behavior further improvement in stem cell transplantations will be achieved.

## 1. Stem Cells

Stem cells are primitive cells that have 3 major characteristics. First, stem cells have a certain potency allowing them to differentiate towards multiple cell types. Second, stem cells have the ability to self-renew meaning they can undergo numerous cell cycles while maintaining their differentiation potency. Third, stem cells can functionally reconstitute a tissue* in vivo* [1]. These unique features make them attractive candidates for the field of regenerative medicine.

In this review, we have focused on adult stem cells because they have already been shown to be safe in clinical trials. We will more specifically discuss neural stem cells (NSCs), mesenchymal stem cells (MSCs), satellite cells (SCs), and mesoangioblasts (MABs) since all of them have been evaluated for therapeutic potential in neurodegenerative and neuromuscular disorders.

First it was thought that NSCs play an essential role during the development of the central nervous system (CNS) until it was terminally differentiated during adulthood [2]. In the last 2 decades several studies discovered that NSCs are still present inside the adult CNS [3]. They have been demonstrated to release beneficial cytokines in the regeneration and repair of neural tissues but also to differentiate* in vitro* and* in vivo* into diverse neuronal lineages and to form networks with surrounding neuronal cells [4, 5].

MSCs represent a very small fraction of bone marrow (0.001%–0.01%) and were first isolated from bone marrow by Friedenstein et al. in 1968 [6]. They have shown to differentiate towards several cell types, including adipocytes, chondrocytes, osteoblasts, and fibroblasts and more recently Woodbury et al. achieved neuron-like differentiation of MSC [7, 8]. Besides isolation from the bone marrow, MSCs have been isolated from almost every tissue and can be readily expanded* in vitro* [9]. Furthermore, MSCs lack immunogenicity and even reduce inflammation and suppress T-cell proliferation [10]. MSCs exert the majority of their effects via their immunomodulatory, neurotropic, and repair-promoting properties. Their effect has been assessed in numerous disease models, including neurologic diseases, and has even reached translation towards clinical trials [11–13].

SCs are located in the periphery of the skeletal myofibers. In mature muscles SCs remain quiescent but following muscle injury they regain mitotic activity and are able to repair the incurred muscle damage [14]. These cells and their derivatives are therefore highly explored for treating several muscle disorders; for a detailed review see Berardi et al. [15].

MABs are vessel-associated stem cells, which were initially isolated from the fetal aorta but are now readily isolated from postnatal vessels of skeletal muscle or heart [16]. They are capable of differentiating towards cell types of the mesodermal lineages, namely, adipocytes, chondrocytes, osteoblasts, and fibroblasts like MSCs [17]. In contrast with MSCs however, MABs differentiate with high efficiency towards myofibers both* in vitro* and* in vivo* following transplantation in dystrophic animals [18].

## 2. Stem Cell Therapies in Neurodegenerative and Neuromuscular Disorders and Acute Injuries

Neurodegenerative and neuromuscular disorders are the consequence of progressive and irreversible cell loss in the human body. Neurodegenerative disorders, like Parkinson's disease (PD) and Huntington's disease (HD), are caused by progressive loss of neurons and mainly impair cognitive function. Neuromuscular disorders can be caused either by motor neuron loss (amyotrophic lateral sclerosis; ALS) or by loss of the actual muscle cells, with Duchenne muscular dystrophy (DMD) as most prevalent example. Furthermore, acute neuronal injuries (spinal cord injury (SCI) and traumatic brain injury (TBI)) also can result in permanent cell loss due to the limited regenerative potential of NSCs. In all these disorders the endogenous stem cells are exhausted and cannot compensate this progressive cell loss. To date no curative treatment has been developed for these disorders.

The fact that stem cells compensate normal tissue turnover, release beneficial paracrine molecules, and are readily isolated and expanded* in vitro* makes them attractive tools for regenerative medicine [19]. We will briefly discuss the different stem cell therapies performed in several neurodegenerative and neuromuscular disorders.

### 2.1. Stem Cell Therapy in Neurodegenerative Disorders

#### 2.1.1. Huntington Disease

Huntington's disease (HD) is caused by a repeated trinucleotide (CAG) within the Huntingtin gene and results in choreiform movements, limb incoordination, and impaired motor function. These choreiform movements are the consequence of death of the medium spiny neurons (MSN) in the caudate, putamen, and globus pallidus [20]. Several groups demonstrated improved motor function after NSCs transplantation [21–25]. In one HD patient who died from cardiovascular disease 18 months after transplantation of neuroblasts, postmortem histological analysis demonstrated surviving transplanted cells with striatal-like morphology without apparent immunological rejection [26].

#### 2.1.2. Parkinson's Disease

Parkinson's disease (PD) is a neurodegenerative disorders caused by the selective death of dopamine-producing neurons in the substantia nigra. In the early stage of the disease the symptoms are mainly movement-related including tremor, rigidity, and bradykinesia [27]. In the later stage, cognitive impairment is also observed.

At the end of the 20th century, several open-label noncontrolled clinical trials were performed using human fetal dopaminergic neurons to replace the loss of dopaminergic neurons in PD patients. These studies demonstrated a mild recovery of motor function and higher presynaptic dopaminergic function detected with positron emission tomography (PET) showing higher uptake of ^18^F-fluorodopa [28, 29]. However, these improvements were not seen in all patients, and two large randomized double-blind clinical trials with neural grafts showed no clinical efficacy [29–31]. These contrasting results have resulted in a large debate if neural transplants could be effective in treating PD patients. One part of the field believes that the beneficial effects seen in the first studies are the consequence of placebo and nonblinded observers, while the other group believes the design of the randomized trials was not optimal [32].

Besides NSCs also MSCs have been evaluated for therapy in PD. The advantage is that they are more readily isolated and expanded* in vitro* than NSCs. Furthermore, differentiation potential towards neuron-like cells and excretion of cytokines and neurotrophic factors has been documented [8, 13]. Starting in 2005, the potential of MSCs in PD was evaluated and protective effects of MSCs on dopaminergic neurons were described [33–35]. The observed beneficial effects of the neurotrophic factor glial cell line-derived neurotrophic factor (GDNF) resulted in studies in which these factors were overexpressed in transplanted MSC [36–38]. Transplantation of these genetically engineered MSCs resulted in functional improvement in PD animal models. Some studies also demonstrated neuronal differentiation of NSCs with increase in the proportion of tyrosine hydroxylase- (TH-) positive and dopamine-producing cells associated with clinical improvements [39–43]. These beneficial preclinical effects have resulted in an open-label study to determine the safety of unilateral transplantation of autologous MSCs in seven PD patients [44]. Only a marginal clinical improvement in three out of the seven patients was observed.

### 2.2. Stem Cell Therapy in Neuromuscular Disorders

#### 2.2.1. Amyotrophic Lateral Sclerosis

Amyotrophic lateral sclerosis (ALS), also known as Lou Gehrig's diseases, is a severe and very rapidly progressive neurodegenerative disorder characterized by degeneration of motor neurons followed by loss of neuromuscular interaction resulting in muscle atrophy with the associated progressive muscle weakness, dysphagia, spasticity, and ultimately death.

MSCs transplantation following different transplantation routes in rodent ALS animal models resulted in a significant delay in ALS disease onset, amelioration of the pathophysiology, and increased survival rate [45–50]. These beneficial effects have been attributed to the neuroprotective effects of MSC, reduced inflammation, and some transdifferentiation towards healthy astrocytes [45–47]. The observed preclinical improvement has led to several phase I studies demonstrating the safety and feasibility of MSC treatment in ALS patients [51–53]. However, the patient cohorts were too small to evaluate efficacy.

#### 2.2.2. Duchenne Muscular Dystrophy

Duchenne muscular dystrophy (DMD) is a severe progressive muscle disorder caused by mutations in the dystrophin gene located on the X-chromosome. The associated muscle damage activates resident endogenous primary muscle stem cells, namely, SCs [54, 55]. However, endogenous stem cells also contain this mutation and have limited self-renewal capacity. Therefore, an inefficient muscle repair process occurs with associated fatty acid depositions and muscle fibrosis. Despite the inefficient regenerative process, the discovery of increased SC proliferation forms the basis of current stem cell therapies for DMD. At present, researchers have found beneficial effects after intramuscular injection of different types of dystrophin expressing myogenic progenitor cells (myoblasts, SC) [56–60]. These beneficial effects have led to a clinical trial of autologous transplantation of CD133^+^ cells, human muscle-derived stem cells with myogenic potential, in 8 boys with DMD [61]. As with previous stem cell transplantations, no adverse events occurred. However, no functional improvement was observed. The absence of functional improvement is caused by insufficient migration of the myoblasts, immunological clearance, and death of myoblasts after transplantation [62–64].

The disadvantage of using myogenic progenitor cells is the inability to migrate over a long distance and through the vascular endothelial wall. This prevents systemic administration of these cells and hampers their clinical applicability. Therefore, in recent years MABs have been studied to treat dystrophic muscles. The advantage of MABs is the ability to pass the endothelial wall of the vasculature and different studies have demonstrated regenerating muscle architecture with functionality after intra-arterial injection both in dystrophic alpha sarcoglycan- (*Scga-*) null mice and in golden-retriever muscular dystrophy (GRMD) dogs [65, 66]. These promising preclinical studies have resulted in a phase I/II clinical trial in 5 DMD patients [67]. The infusion of the cells was relatively safe; one patient however developed a thalamic stroke without clinical consequences. No functional improvement could however be observed and very low level of donor DNA was retrieved in muscle biopsies of the patients.

### 2.3. Stem Cell Therapy in Acute Injuries

#### 2.3.1. Traumatic Brain Injury

Traumatic brain injury (TBI) is becoming increasingly important as indicated by its rapid increase in incidence in different countries [68]. Furthermore, TBI is a well-established risk factor for different neurodegenerative diseases [69]. NSCs improved metabolic recovery and neurological motor function in a TBI rat model [70]. MSCs have shown to elicit neuroprotective and regenerative effects after administration in TBI animal models [71, 72]. Contrasting studies which showed no improvement have also been reported [73].

#### 2.3.2. Spinal Cord Injury

Spinal cord injury (SCI) occurs after traumatic damage to the spinal cord which is associated with severe consequences and might even result in death [74]. The transplantation of NSCs in SCI models resulted in astrocytic and neuronal differentiation and improved remyelination, motor function, and sensory perceptions [75–83]. Another study also found glial and neuronal differentiation; however no functional improvement could be found [84].

MSCs have demonstrated to have beneficial effects in models of SCI and their effect is mediated through reduced inflammation, improved angiogenesis, suppression of neuroinhibitory molecules, reduced demyelination, and induction of remyelination [85–98]. The paracrine factors involved in these positive effects were brain-derived neurotrophic factor (BDNF), GDNF, nerve growth factor (NGF), leukemia inhibitory factor (LIF), and insulin-like growth factor-1 (IGF-1) [91, 99, 100]. MSC treatment in patients with SCI was shown to be safe, with some modest improvement in clinical scores [101–104].

## 3. Stem Cell Imaging

Despite the progress into clinical trials, limited information is available on the distribution, migration, and survival of these cells in living organisms over time [105]. At present, histology is the golden standard in preclinical cell monitoring. However, this requires the sacrifice of numerous animals and can only be obtained in a very limited manner in clinical trials, hampering clinical translation. Furthermore, it provides no longitudinal or whole body monitoring. This results in a shortage of information on stem cell behavior* in vivo*. Therefore, noninvasive cell monitoring methods have been developed [106–108]. This provides direct visualization of stem cell delivery together with an indication of the location of the transplanted cells and their survival over time.

**Figure 1:**
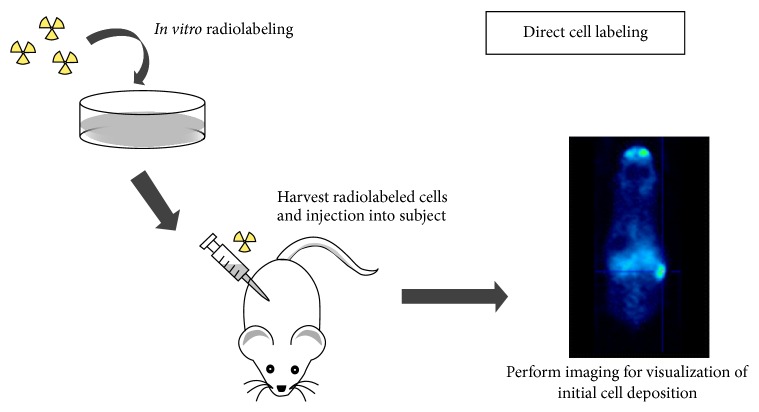
Schematic overview of the processes involved in direct cell labeling. Cells are first labeled with contrast agents* in vitro* through incubation, harvested, and injected into a subject (adapted from [106]).

Stem cell imaging approaches can be divided into direct and indirect cell labeling. Direct cell labeling is the most frequently used method and consists of labeling cells* in vitro* with reporter probes, including fluorophores, radiotracers, or paramagnetic nanoparticles (NP), by incubation before transplantation (Figure 1) [106, 109]. These reporter probes can either bind to specific epitopes on the cell membrane, like a copper-64-labeled antibody or zirconium-89-desferrioxamine-NCS (^89^Zr-DBN) [110, 111], or be taken up by passive diffusion or transporters, like indium-111- and ^89^Zr-oxine or 2-[^18^F]-fluoro-2-deoxyglucose (^18^F-FDG) [112, 113]. After incubation, the cells are injected* in vivo* for cell monitoring by optical imaging, single photon emission computed tomography (SPECT), positron emission tomography (PET), or magnetic resonance imaging (MRI).

The direct cell labeling procedure is straightforward and this is the major advantage of direct labeling. However, for radionuclide imaging this labeling strategy is limited to short-term cell visualization based on the half-lives of the tracers (*t*_1/2_ fluorine-18 = 110 min; *t*_1/2_ indium-111 = 67.32 h; *t*_1/2_ zirconium-89 = 78.41 h) [118, 119]. Also, the proliferation of directly labeled cells results in the dilution of the reporter probe preventing the visualization of daughter cells and compromising the correlation of the observed imaging signal with cell amount. Another disadvantage, mainly for NP, is the persistence of the probe after cell death, which results in reporter probe transfer towards adjacent phagocytic cells like macrophages and microglia. This prevents correct correlation between the obtained image signal and cell viability [120, 121].

**Figure 2:**
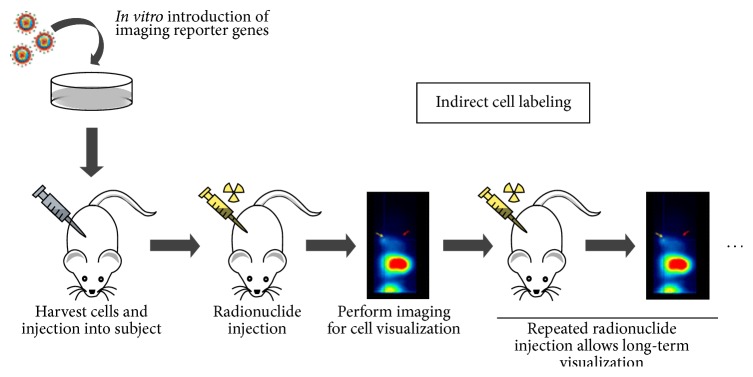
Schematic overview of the steps involved in indirect cell labeling. First, imaging reporter gene expression is induced* in vitro* in host cells. Reporter gene expressing cells are harvested and injected into a subject. In a next step, the respective radiotracer is injected and imaging can be performed to determine the localization of the cells. Repetitive injection of the radiotracer allows for repetitive imaging and thus long-term visualization of the engrafted cells [106].

Indirect cell labeling consists of cellular modification by inserting an exogenous reporter gene into cells (Figure 2). For stem cell monitoring, the reporter gene is inserted into the stem cell before transplantation [106]. Reporter genes are genes that encode proteins that can be detected by noninvasive imaging, either directly or because they bind a signal generating ligand.

Nuclear imaging reporter genes are transporters (e.g., sodium iodide symporter (NIS)), receptors (e.g., dopamine receptor type 2 (D_2_R) or somatostatin receptor type 2), or enzymes (e.g., herpes simplex virus type 1 thymidine kinase (HSV1-tk)) for which radiolabeled ligands have been developed. After transfer of the reporter gene and its transcription and translation into a protein, the cells are able to bind, accumulate, or convert an exogenously given reporter probe. This results in specific uptake of the reporter probe in reporter gene-expressing cells and allows long-term longitudinal noninvasive visualization of cells* in vivo*. Indeed, after decay of the reporter probe (e.g., after 5 half-lives) a novel administration can be performed and the distribution of the reporter probe will reflect reporter gene expression at that time point. Furthermore, the accumulation of the reporter probe is proportional to the total gene expression and thus to the total cell amount.

Because only viable cells translate the reporter gene into a protein, a distinction between viable and nonviable cells can be made [107]. The disadvantage of this technique is the insertion of genomic material into a cell which might disrupt normal cell physiology [122, 123]. However, with novel site-specific gene editing approaches the reporter genes can become integrated in a safe harbor locus [124, 125].

## 4. Imaging Studies Performed with Stem Cells in Healthy Animals

### 4.1. Direct Cell Labeling

One of the most frequently used direct cell labeling approaches is via the use of superparamagnetic iron oxide (SPIO) NP which generate a hypointense contrast on MRI. Several aspects are important when designing SPIOs for cell labeling. The SPIOs need to be readily taken up by the cell and have a high T2 relaxivity to generate contrast, long-term retention in the cell and biocompatibility with the cell and host (for detailed review see Li et al. [126]). Successful labeling of NSCs [114, 127, 128], MSCs [129–133], SCs [129, 134, 135], and MABs [136] with SPIO NP has been demonstrated by different groups and shown to be without any consequence on viability, differentiation capacity, or biological characteristics. Cromer Berman et al. however observed an impairment of NSC motility and migration, both* in vitro* as* in vivo* [137].

**Figure 3:**
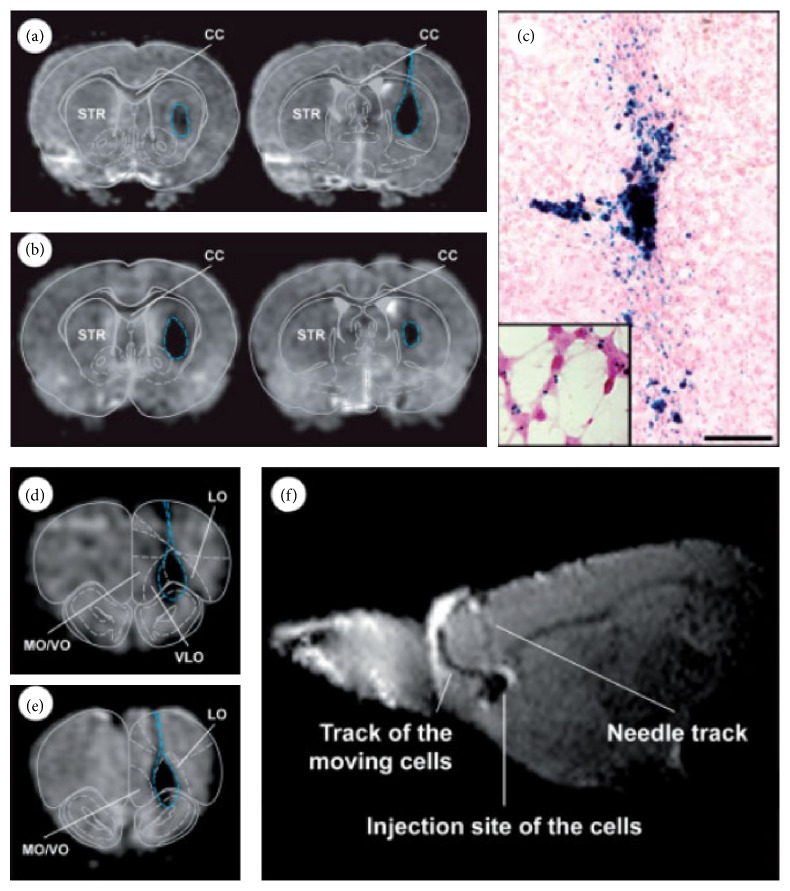
Longitudinal MRI studies of transplanted SVZ cells. T2-weighted MRI studies of transplanted SVZ cells 2 h after transplantation into the striatum (a) and 21 days later (b). Images were acquired with a 9.4 T machine. The needle track and cell transplant have been arbitrarily delineated by a dotted blue line. The schematic atlas drawings have been overlaid onto MRI images to facilitate anatomical localization of the graft site (see methods for overlay procedure). (c) Prussian blue staining of the rat striatum depicts distribution of transplanted, iron oxide-labeled SVZ cells one month after transplantation. Iron deposits stain blue with potassium ferrocyanide. Cells are also counterstained with pararosaniline allowing the cytoplasm to appear as light pink also depicted in cells* in vitro* (see inset (c)). Further MRI of SVZ cells into the RMS confirmed correct positioning of the graft immediately after the transplantation procedures (d) and normal localization and growth 14 days after transplantation (e). (f) A sagittal T2-weighted MR image shows the migration of SPIO-labeled SVZ cells towards the olfactory bulb 7 days after transplantation. STR, striatum; CC, corpus callosum; LO, lateral orbital cortex; VLO, ventrolateral orbital cortex; MO/VO, medial orbital cortex/ventral orbital cortex [114].

Cicchetti et al. were able to demonstrate via MRI rostral migratory stream (RMS) migration of transplanted SPIO-labeled NSCs (Figure 3) [114]. This was associated with enhanced expression of D_2_R and dopamine transporter as indicated by increased ^11^C-raclopride and ^11^C-CFT binding, respectively. Afterwards immunohistochemistry demonstrated that this was the consequence of host-derived changes because the transplanted cells were still at an early stage of development.

The disadvantage of SPIO-based cell labeling is the possibility of aspecific uptake of the NP by macrophages and microglia. Berman et al. combined SPIO-labeling with indirect cell labeling via NSCs expressing firefly luciferase (Fluc) to monitor cell survival in immunocompetent and immunodeficient animals [138]. Only in immunodeficient animals long-term cell monitoring via bioluminescence imaging (BLI) was achieved. In contrast, hypointense SPIO signals on MRI were persistent both in immunocompetent and in immunodeficient animals. Histology demonstrated that even in the immunodeficient animals no colocalization of the SPIOs with the NSCs occurred and that the particles were taken up by microglia (the resident immune cells of the CNS). Therefore, caution needs to be taken into account for the interpretation of SPIO-based imaging results.

Other disadvantages of SPIO-based cell monitoring are the low specificity due to other organs with hypointense signal and the complicated* in vivo* quantification of the signal loss.

Therefore, recently an alternative MRI-based method has been evaluated, namely, ^19^F MRI, which detects the stable isotope fluorine-19. In contrast to SPIO-based cell monitoring, ^19^F MRI is able to image implanted cells with high specificity due to the lack of detectable fluorine signal in biological tissue [139, 140]. Furthermore, quantification of the implanted cells is possible since the ^19^F MRI signal intensity is linearly related to the number of ^19^F-labeled cells. Successful labeling of NSCs [140] and MSCs [141] with ^19^F NP was achieved without altering cell characteristics. No leakage of the NP towards surrounding macrophages was observed in ^19^F-labeled NSCs. However, the ^19^F contrast used for labeling of MSCs was localized in macrophages instead of the grafted cells. Therefore, also via this approach the observed imaging results might not correspond with the injected cell population.

Radionuclide imaging techniques are highly quantitative in nature and therefore several groups have directly labeled NSCs and MSCs with either ^18^F-FDG [142], ^99m^Tc-HMPAO [143–146], or ^111^In-oxine [147–149]. After labeling with radionuclides no differences in viability or differentiation capacity were shown; no ultrastructural changes were shown either [111, 142–144, 147–149]. ^18^F-FDG did also not induce metabolic changes while ^111^In-oxine reduced metabolic activity and motility of the cells [148]. A reduced proliferation rate was also seen in ^99m^Tc-labeled NSCs, probably because of DNA damage induced by Auger electrons [146].

All previous radionuclides have a relatively short half-life (*t*_1/2_^18^F = 110 min; *t*_1/2_^99m^Tc = 6.03 h; *t*_1/2_^111^In = 67.32 h) and are therefore restricted to short-term cell monitoring with 5 half-lives as upper limit. Furthermore, relative high efflux rates are observed with some of these radionuclide labeling approaches [142, 150]. Bansal et al. developed a ^89^Zr-DBN labeling agent which is capable of covalently binding to the cell surface and allows cell monitoring for 2 to 3 weeks because of the long half-life (78.41 h) [111]. Seven days after intravenous injection of ^89^Zr-labeled MSCs in healthy athymic mice, cells could mainly be detected in the lungs (50 ± 27%). The remainder was found in the liver (27 ± 19%) and the bones (16 ± 5%). In an acute myocardial ischemia model, ^89^Zr-labeled MSCs were injected in the ischemic area of the heart. Seven days after delivery, 20 ± 7% of the cells were maintained in the heart while the other fraction was distributed to the lungs (40 ± 16%), bone (29 ± 11%), and liver (7 ± 5%).

Both MRI and PET have a temporal resolution in the order of several seconds to minutes, which precludes them from being used during the cellular implantation procedure. Photoacoustic imaging (PAI) could however be used because of its high temporal resolution [151]. To allow monitoring of MSCs with PAI, cells were labeled with silica-coated gold nanorods. After intramuscular transplantation, successful monitoring at high temporal resolution (0.2 s) could be achieved with PAI until day 4. The lowest cell amount to be monitored was 1.0 × 10^5^ cells.

### 4.2. Indirect Cell Labeling

All the labeling approaches mentioned above are limited to short-term cell monitoring (hours to weeks) or might generate aspecific signals. As an alternative, reporter gene-based strategies have been developed. Not only does it allow long-term noninvasive imaging of stem cells, it also makes a distinction between viable and nonviable cells. The most widely used imaging reporter gene is Fluc which catalyzes the oxidation from D-luciferin to oxyluciferin with the release of visible light (principle of BLI) in the presence of oxygen, magnesium, and adenosine triphosphate.

**Figure 4:**
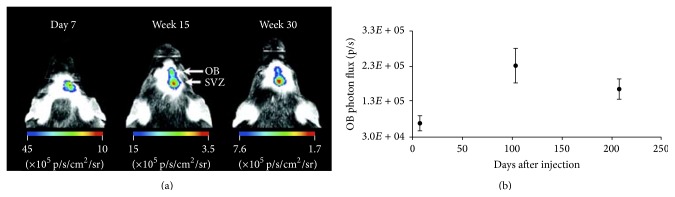
*In vivo* long-term follow-up of stem cell migration with bioluminescence imaging (BLI). (a) At day 7 after injection there was a detectable BLI signal only at the site of injection (SVZ). At 15 and 30 weeks after injection an additional focus was detected at the OB projection site, as well as the original focus at the SVZ. A representative mouse is shown. (b) The graph shows the quantification of the* in vivo* OB BLI signal from all the animals followed up for 30 weeks (*n* = 14). The OB photon flux at weeks 15 and 30 was significantly higher than that on day 7 (*p* = .002 and .045). OB, olfactory bulb; p, photons; s, second; sr, steradian; SVZ, subventricular zone [115].

Endogenous cells of the subventricular zone have been labeled with enhanced green fluorescent protein (eGFP) and Fluc via a lentiviral vector. Afterwards the migration of NSCs towards the olfactory bulb (OB) via the RMS could clearly be observed via BLI (Figure 4) [115]. A strong linear correlation between the* in vivo* OB photon flux and number of eGFP-positive cells on histology was shown. This allowed us to evaluate the effect of BDNF of neurogenesis of endogenous stem cells. Short-term stimulation with BDNF (1 month or less) demonstrated increased neurogenesis; in contrast however long-term overexpression impaired migration and differentiation of NSCs as indicated via BLI [115].

Incorporation of Fluc in NSCs [138], MSCs [152, 153], and SCs [154] has been successfully performed for cell monitoring with BLI. This noninvasive read-out of cell survival was used by Liang et al. to demonstrate that human NSCs expressing Fluc had a significant increase in cell survival when coinjected with helper cells expressing basic fibroblast growth factor (bFGF) in the striatum [155]. Stem cell therapy can also be combined with gene therapy; to visualize both cell survival and therapeutic gene expression Gheysens et al. transduced myoblasts with Fluc and HSV-tk to monitor cell survival and transfected the cells with Renilla luciferase (Rluc) and the therapeutic gene (hypoxia-inducible factor-1*α* fused to two repeats of VP16; HIF-1*α*-VP2) [154].* In vitro*, a clear linear correlation was found between HIF-1*α*-VP2 and Rluc activity. Furthermore, they demonstrated that the recombinant hybrid HIF-1*α*-VP2 protein could effectively induce downstream angiogenic gene expression, indicated by the amount of vascular endothelial growth factor (VEGF) and placental growth factor (PIGF) in the supernatant of the cells. After intramuscular injection of myoblasts expressing Fluc and myoblasts expressing Fluc combined with Rluc and HIF-1*α*-VP2 (left and right leg, resp.) robust Fluc-mediated signal was detected in both legs immediately after cell implantation, whereas RLuc signal was only observed in the right leg. Therefore, they were able to monitor transplanted cell survival and therapeutic transgene expression, which is crucial for evaluating combined cell and gene therapy.

Although BLI is a valuable and widely used tool for preclinical cell monitoring, it can never be translated towards a clinical setting. Therefore, different PET and/or SPECT [156–163] and MRI [163–167] reporter genes have been developed.

Endogenous neuronal cells have been labeled with the MRI reporter gene ferritin. Ferritin is capable of taking up iron and generates a hypointense (lack of) signal on MRI. Stable transduction of the cells in the striatum was feasible, and when no inflammation was present clear MRI signals could be observed [166]. However, low sensitivity was observed if there was ongoing inflammation because ferritin is highly expressed on reactive microglia [168]. Vande Velde et al. evaluated the feasibility to monitor endogenous migration of cells in the SVZ with ferritin as reporter gene [167]. Clear hypointense regions on MRI were present in the transduced area; however monitoring the RMS towards the OB via* in vivo* MRI was not feasible because of low sensitivity. High resolution* ex vivo* MRI was capable of quantitatively detecting hypointense regions in the OB; but based on visual assessment this was not feasible. The divalent metal transporter 1 (DMT1) is a reporter gene that can be used for both MRI and PET [163]. Monitoring of human NSCs expressing DMT1 after injection in the striatum was feasible with MRI after manganese injection; ^52^Mn-based PET/CT was unable to visualize the graft although* ex vivo* results demonstrated low levels of brain uptake.

Also radionuclide reporter genes, namely, the D_2_R [160] and the human NIS [161, 162], were used for labeling of exogenous MSCs. Since binding of the ligand on wild-type D_2_R activates a signaling pathway, a mutant variant, the so-called D_2_RB80A, was developed with similar* in vitro* and* in vivo* binding activity [160]. No long-term cell monitoring was achieved with MSCs transduced with a baculovirus encoding hNIS; however this was the consequence of transient reporter gene expression via the baculovirus approach [161, 162].

To benefit from the different imaging approaches reporter genes were also combined to provide a better understanding of the localization, survival, distribution, and differentiation processes of stem cells* in vivo*. We combined Fluc with hNIS in a lentiviral construct and this allowed us to monitor MSCs via BLI, Cherenkov luminescence imaging, and PET [169]. Pei et al. combined Fluc with another radionuclide reporter gene (HSV-tk) and successfully monitored the cells with BLI and PET [170].

In the aforementioned studies only information on the location of the cells is provided. To visualize the true differentiation capacity of the cells noninvasively Hwang et al. developed a dual reporter gene system, expressing both Fluc and NIS under control of the neuron-specific enolase (NSE) promoter [171]. During neuronal differentiation of transfected F11 cells, a cell line derived from dorsal-root-ganglion neurons, a 4-fold increase in luciferase and NIS expression was observed.* In vivo*, the differentiation of F11 cells towards neurons could only be visualized with BLI but not with SPECT [171]. In a different study, the impact of neuronal activator neurogenin 1 (Ngn1) on neuronal differentiation was assessed in F11 cells expressing Fluc under a neuronal promoter [172]. After subcutaneous and striatal injections an increase in luciferase activity was observed in F11 cells cotransfected with Ngn1 compared to sham transfected F11 cells. These studies implicate the strength of imaging reporter genes to visualize differentiation of cells* in vivo*.

## 5. Imaging Studies Performed with Stem Cells in Disease Models and Patients

### 5.1. Huntington Disease

**Figure 5:**
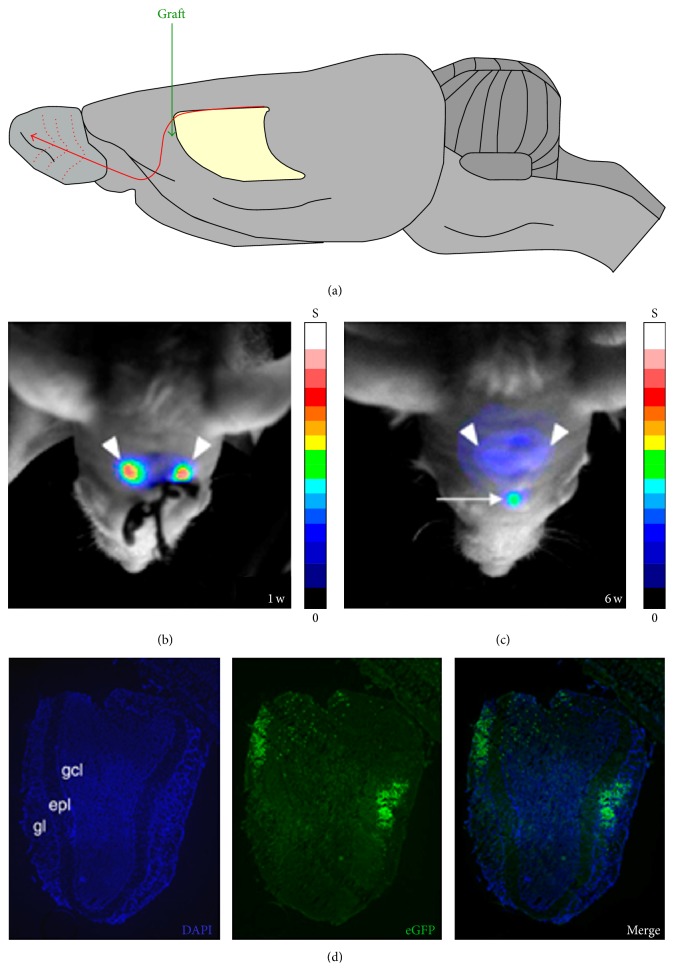
Tracking of NSC migration by* in vivo* optical neuroimaging. (a) Diagram showing the rostral migratory stream of endogenous NSCs, from the subventricular zone to the olfactory bulb. (b) Optical neuroimage of c17.2/Luc-NSCs transplanted in the mouse rostral migratory stream, acquired 1 week after grafting. (c) Bioluminescent spot (arrow) detected over the olfactory bulb of the same mouse 6 weeks after grafting. The arrowheads indicate the grafting sites. (d) Histological analysis showing eGFP-positive c17.2/Luc-NSCs in the olfactory bulb. Counterstaining with DAPI (blue) shows the presence of eGFP-expressing cells in the glomerular and granular cell layers, the natural migration target for NSCs of the subventricular zone (*n* = 3) [116].

A single cell monitoring study has been performed to visualize stem cells in HD animal models. NSCs expressing Fluc were injected in the brain and clear RMS migration of the NSCs towards the OB was observed via BLI (Figure 5) [116]. These results are in agreement with the labeling of endogenous stem cells and indicate that transplanted exogenous cells are able to follow similar migration routes [115]. To also assess the effect of GDNF, NSCs were also transduced to overexpress GDNF. A Huntington disease mouse model was generated in which the lesion site had a reduction of 86% in striatal neurons compared to the nonlesion site. When Fluc expressing NSCs were transplanted higher cell proliferation was seen in the lesion site compared to the nonlesioned striata; however no recovery in neuronal death was observed when Fluc expressing NSCs were transplanted. In contrast mice receiving Fluc and GDNF expressing NSCs at the lesion site had mild reduction in neuronal death, which demonstrated the neuronal protective effects of GDNF. This was further confirmed via behavioral testing, where a reduction in asymmetric behavior was observed in HD mouse model after treatment with NSCs expressing GDNF.

### 5.2. Parkinson's Disease

To generate more insight in MSC-mediated therapy in PD, Guo et al. labeled MSCs with SPIOs. Different amounts of SPIO-labeled cells were injected in the right striatum of a 6-hydroxydopamine (6-OHDA) PD rat model, which is a widely used model to mimic PD. This resulted in clear dose-dependent hypointense signal at the site of injection for 12 weeks [173]. Extensive migration of transplanted cells towards the lesion site was only detectable on histology. In contrast to the MRI images, only a few iron-labeled MSCs were found in the striatum where the hypointense signal was located on the MRI scans. Furthermore, they observed an increase in dopaminergic glial fibrillary acidic protein (GFAP), NSE, and nestin following transplantation, which suggest that either the transplantation enhanced the differentiation of resident stem cells or some of the transplanted cells had differentiated.

Im et al. demonstrated via BLI that there is no difference in NSCs survival when transplanted in the right striatum of 6-OHDA mouse model or healthy mice [174]. In both animal groups cells were lost 10 days after injection. Behavioral testing demonstrated significant increase in asymmetric behavior in the 6-OHDA mouse model. This was further confirmed by reduced binding of ^18^FN-(3-fluoropropyl)-2′-carbomethoxy-3′-(4-iodophenyl)nortropane (^18^FP-CIT), which binds the dopamine transporter, in the right striatum of the 6-OHDA mouse model. At 1 week after injection no difference in cell- and sham-injected groups could be observed. At 4 weeks after injection however, a significant reduction in asymmetrical behavior was shown in cell-treated animals. On PET no improvement in striatal binding potential (BP) ratio after cell transplantation was observed in the 6-OHDA mouse model group. Histology demonstrated that the majority of the transplanted cells died in the early phase of transplantation with only a small fraction of cells capable of surviving. However, this was below the detection limit of BLI.

### 5.3. Amyotrophic Lateral Sclerosis

To increase the understanding of potential mechanism of MSC therapy in ALS, Canzi et al. labeled skeletal-derived MSCs with SPIOs and injected them intracerebroventricularly in a murine model of spontaneous motor neuron degeneration (the Wobbler mouse) [175]. MRI showed hypointense signals throughout the ventricular system one day after cell transplantation and this signal decreased but remained detectable until 14 weeks after transplantation. Based on immunostaining only modest human MSCs integration in the brain parenchyma could be demonstrated. To validate the clinical improvement, cell transplantations were performed without cell labeling. This however prevents the correlation of functional effects with the imaging results. Furthermore, no assessment of the labeling procedure on the beneficial effects of the cells could be made. The nonlabeled cells improved the forepaw atrophy and grip strength. This might be caused by a modest improvement in the percentage of active neuromuscular junctions and a mild attenuation of microglial activation. The authors attributed the effects to the release of the anti-inflammatory cytokines interleukin-10 (IL-10) and IL-13 by the transplanted MSCs. These cytokines were however not able to reduce motor neuron death in these Wobbler mice as indicated by histology.

### 5.4. Duchenne Muscular Dystrophy

To evaluate the* in vivo* behavior of myoblast progenitors overexpressing mini dystrophin Cahill et al. labeled them with SPIOs [176]. SPIO-labeled cells were injected in the posterior leg musculature and a hypointense signal could be observed on MRI at all-time points (end point day 14). Engrafted cells were detected by histology and dystrophin expressing fibers with iron content were found. A small number of macrophages, which are frequently present in regenerating fibers, also contained iron. To evaluate the transfer of SPIOs after cell death towards other cells human SPIO-labeled MSCs were injected into immune competent mice. A slow decrease in SPIO-induced contrast over time was observed. These results indicate that it may require at least 2 months following an intramuscular injection before large amounts of residual SPIOs are removed. Because all the striated muscles in the human body are affected by DMD, systemic cell administration strategies have been evaluated. After intra-arterial injection SPIO-labeled myoblast progenitors were visualized in the vasculature of the muscle via MRI [176]. Histology confirmed the presence of labeled cells within the arteries and capillaries but not inside the muscle fibers. In contrast to intramuscular injection, intra-arterial injection resulted in rapid clearance of the cells 3 days after transplantation.

In contrast to myoblast progenitors, MABs are capable of crossing the vessel endothelial wall. Therefore, also MABs were labeled with SPIOs [136]. If these cells were injected in the gastrocnemius of mdx mice, which is a DMD animal model, cells were clearly visible 24 h following delivery and remained visible in the following 2 weeks. SPIO-labeled MABs were also injected in the left ventricular wall of the heart and remained visible until 6 months after injection. Histology demonstrated that all detectable iron-labeled cells were however proinflammatory macrophages which clearly confound the observed imaging results. Some MABs may have survived since low levels of dystrophin were detectable in cell-injected animals, although there is always the risk of revertant fibers by spontaneous mutation restoring the nonsense mutation in the dystrophin gene. Since DMD affects striated muscles throughout the body, systemic delivery of stem will be necessary. Therefore, SPIO-labeled MABs were injected in the left ventricle of mdx mice. After 5 days, cells were not detectable in filter organs or in muscle tissue via MRI. Postmortem analysis however indicated that iron-labeled cells had localized to dystrophic skeletal muscles and vessels in cardiac muscle.

To understand more about MABs survival and the impact on immune suppressants on cell survival, we transduced MABs with Fluc and hNIS [177]. MABs expressing Fluc and hNIS were intra-arterially injected in* Sgca* ^−/−^ mice, which is a dystrophic animal model, and visualized via both BLI and PET. Based on noninvasive imaging data we were able to demonstrate that costimulation adhesion blockade therapy was superior to cyclosporine A in reducing cell rejection. T-cell analysis showed us that costimulation adhesion blockade therapy was capable of reducing the number of cytotoxic T-cells and upregulating the regulatory T-cells. Although costimulation adhesion blockade therapy was a superior immune suppressant it was not able to achieve long-term cell survival with day 21 after injection as latest time point to visualize the cells. In the MAB-treated animals with costimulation adhesion blockade as immune suppression a transient improvement in running distance was observed together with mild reexpression of alpha sarcoglycan.

### 5.5. Traumatic Brain Injury

To improve the understanding of MSC-based therapy in TBI, several noninvasive MSC tracking studies have been performed. SPIO-labeled MSCs could be visualized after intracranial injection via MRI in brain lesion models [132, 178]. When a lesion was present MSCs migrated preferentially towards the lesion site. Jackson et al. were able to visualize this migration by MRI while Delcroix et al. were only capable of showing this via histology [132, 178]. To avoid the invasive intracranial administration procedure, systemic administration routes have been evaluated either with directly labeled MSCs using SPIOs for MRI [132, 133] or with radionuclides (^99m^Tc-HMPAO and ^111^In-oxine/tropolone) [144, 149]. Again, migration towards the lesion site was observed and could be visualized via MRI and SPECT [132, 133, 149]. Park et al. were unable to visualize ^99m^Tc-HMPAO-labeled MSCs in the brain; however* ex vivo* uptake showed increased uptake in the lesion site compared to the contralateral site [144]. Huang et al. further demonstrated that specific SPIO design clearly upregulated CXCR4 expression [133]. The overexpression of CXCR4 resulted in increased cell migration both* in vitro* and* in vivo* in response to SDF-1*α*, the ligand of CXCR4, which is highly secreted after TBI. MSC-treated mice showed signs of decreased contusion volume and less scarring upon postmortem examination and this improvement was higher in SPIO-labeled MSCs compared to nonlabeled MSCs.

**Figure 6:**
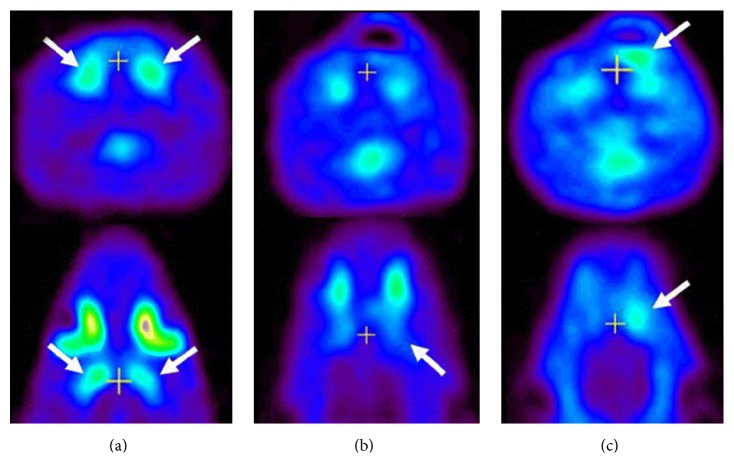
Typical examples of ^11^C-NMSP micro-PET imaging in various conditions. Coronal and axial sections of rat brain are shown. White cross markers in the coronal and axial sections indicate the same position in a scan. (a) Typical image of ^11^C-NMSP micro-PET in normal rat brain showing high ^11^C-NMSP accumulation in striatum. (b) L/N ratio of ^11^C-NMSP decreased after traumatic brain injury. (c) High accumulation of ^11^C-NMSP indicated the existence of transplanted DRD2-positive NSCs [70].

To assess cell survival after transplantation in a rat model of TBI, NSCs were* in vitro* exposed to BDNF containing media to induce D_2_R expression [70]. After transplantation of D_2_R expressing NSC, significant increased binding of ^11^C-NMSP, a specific tracer of the D_2_R, was observed via PET (Figure 6). A continuous decrease in tracer uptake was detected over time; however cell visualization remained feasible 14 days after transplantation. The therapeutic effect of the NSCs was assessed via ^18^F-FDG and showed significant metabolic recovery in cell-treated animals. Also neurological motor function was significantly improved.

One clinical study with SPIO-labeled NSC was performed in a TBI patient. Autologous NSCs were labeled with SPIOs (Feridex®) the day before transplantation and stereotactically implanted around the brain lesion [179]. Pronounced hypointense signal was observed at the injection site which was not present before injection. Clear migration of the signal could be observed around the lesion suggesting NSC migration from the injection site towards the border of the damaged tissue. No hypointense signal was observed after 7 weeks, which might be attributed to a dilution of the signal by cell proliferation or loss in cell survival. To demonstrate that the hypointense signal was not generated by invading macrophages, the experiment was repeated in a rat model of TBI. Histology confirmed the absence of SPIO uptake by macrophages [179].

### 5.6. Spinal Cord Injury

To assess the best administration route for MSCs in rats with SCI, cells were labeled with ^111^In-oxine and administered either intravenously or in the lesion site [180]. The direct injection at the lesion site was clearly superior compared to the intravenous injection since all activity was retrieved in the area of the lesion without any migration towards the other organs throughout the study. In contrast, the intravenous administration resulted in distribution of the cells towards the spleen, liver, and kidneys with hardly any activity in the vertebral column and no activity in the spinal cord. Another study with SPIO-labeled MSCs was however capable of detecting the cells with* ex vivo* MRI in the lesion site after intravenous injection [181]. Furthermore, they demonstrated improvement in Basso, Beattie, and Bresnahan (BBB) locomotor score, heat sensitivity, and lesion size in cell-treated animals. Injection of SPIOs as such resulted in only weak hypointense signal in the lesion site, indicating minimal uptake of the NP by macrophages which was also confirmed by histology.

Also the intrathecal administration route was evaluated for SPIO-labeled MSCs [182]. In this study, they used the SPIOs not only as contrast agent but also as a method to magnetically target the labeled cells. Hypointense signal was higher at the lesion site in the magnetically guided group. The higher cell migration towards the lesion site was associated with increased beneficial effects including axonal integrity and BBB locomotor rating scale. Therefore, magnetic guidance technology for cell delivery might be a promising approach for clinical treatment of SCI.

To have an increased understanding on cell survival after transplantation in mice with SCI Okada et al. transplanted Fluc expressing NSCs in injured spinal cords either during the acute phase (immediately after SCI) or 9 days after the injury (delayed phase) [183]. Drastic reduction in signal intensity was observed the first 4 days after transplantation, which was followed by a relatively stable bioluminescent signal for 6 weeks. No difference in cell survival was observed in cells transplanted during the acute or delayed phase. However, the faith of the transplanted NSCs was clearly different. During the acute phase, cells mainly differentiated towards astrocytic glial scar tissue with only a small percentage differentiating to neurons and oligodendrocytes. In the delayed phase however, neuronal and oligodendrocyte markers were clearly expressed, indicating the importance of the microenvironment on the differentiation of transplanted NSC. In both conditions partial recovery of hind-limb movement was observed within 1 week after transplantation followed by a period of gradual recovery, whereas slower recovery was observed in sham-treated animals.

**Figure 7:**
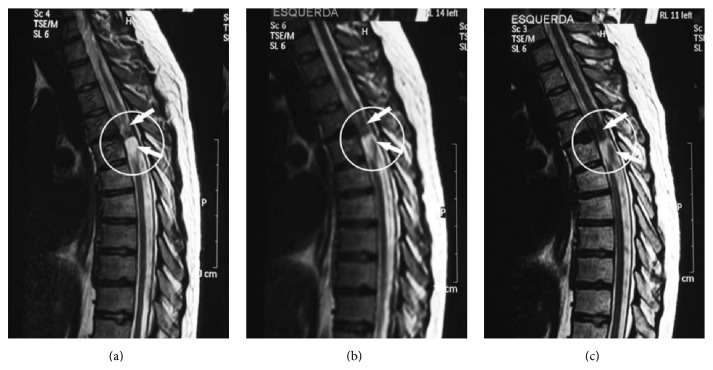
Examples of sagittal resonance images of the spinal cord at the site of the injury, T4-T5 level (white circle) obtained from patient 5 before (a) and 20 and 35 days after labeled-CD34^+^ cells transplantation ((b) and (c), resp.). The suggested CD34 cell migration into the injured site is demonstrated as hypointense areas (black areas) in (b) and (c) (arrows). These hypointense areas are not seen in (a) (arrows) [117].

All the beneficial preclinical effects of stem cell therapy in SCI combined with the added value of noninvasive imaging resulted in two case reports where SPIO-labeled cells were monitored with a clinical imaging system after transfer in SCI patients [117, 184]. Callera and de Melo performed a preliminary safety study to evaluate the possibility to deliver bone marrow precursor cells (CD34^+^ cells) into the spinal cord via an intrathecal transplantation approach in patients with SCI [117]. Twenty days after transplantation, a hypointense signal was specifically visible at the lesion site in 50% of the cell-treated patients (*n* = 10) and the signal persisted until the end of the MRI follow-up period (35 days) (Figure 7). Cell monitoring was feasible if the injected cell number was higher than 0.7 × 10^6^ cells. In the 24-week follow-up period, no adverse events were observed. Chotivichit et al. treated a single SCI patient with SPIO-labeled MSCs [184]. The same injection route was used as in the previous study; however a significantly higher amount of cells (30 × 10^6^ cells) was injected. To achieve this cell quantity cells were maintained in primary culture for four weeks. Immediately after infusion an MRI scan was performed to validate cell injection in the arachnoid space and a hypointense signal was observed in the subarachnoid space and some in the cauda equina but not in the cervical spine cord. Two days later a hypointense signal was observed at the injured cervical spine. The signal was very faint two weeks after transplantation and was not detectable at two and seven months after transplantation. There was no change in spinal cord structure based on MRI. Furthermore, no improvement in neurological deficit was observed with even worsening of neuropathic pain in the patient. Additional adverse events were headache, fever, and transient neurological deficit. These problems were also reported in other studies involving intrathecal stem cell transplantation, with or without stem cell labeling [185, 186].

### 5.7. Skeletal Muscle Injury

To assess the impact of MSCs on skeletal muscle injury cells were labeled with SPIOs. One week after inducing muscle trauma, animals were transplanted with 2.5 × 10^6^ or 5.0 × 10^6^ SPIO-labeled MSC at the lesion site [187]. One day after transplantation, hypointense areas appeared within the traumatized soleus muscle and they remained detectable throughout the investigation period of 65 weeks. No difference in gray scale values was observed between the animals transplanted with 2.5 × 10^6^ or 5.0 × 10^6^ cells. The absence of migration as determined with MRI supports the theory of cytokine release which improves muscle healing. An MRI-based imaging approach was however more complicated because of the hypointense signals observed in normal tissue, such as blood vessels, tendons, and boundary layers between the muscles.

Another study used a BLI-based approach with MSCs isolated from ubiquitously Fluc expressing transgenic rats [188, 189]. Cells were also magnetically labeled to allow guidance towards the injured sites. After transplantation BLI signal could be observed in both the magnetically guided and nonguided group and gradually decreased after day 3 until it was hardly detectable* in vivo* after 4 weeks. Cell survival in the magnetically guided MSCs was higher until 3 days after injection. Although no differences could be observed at later time points via* in vivo* BLI,* ex vivo* BLI showed detectable cell grafts in the muscles 4 weeks after injection and the highest BLI signal was present in the magnetically guided group. Functional recovery, improved revascularization, and reduced fibrosis were observed in all cell-treated animals and these effects were significantly higher in the magnetically guided group.

Since SCs are the endogenous machinery to repair muscle fibers, also SCs were evaluated for their effect in skeletal muscle injuries. Therefore, SCs have been isolated from ubiquitously Fluc expressing transgenic mice. Either freshly isolated SCs or cultured primary myoblasts were injected intramuscularly in nonobese diabetic/severely combined immune-deficient (NOD/SCID) mice depleted of endogenous SC by 18 Gy irradiation [59]. At 4 weeks after injection only freshly isolated SCs produced a robust BLI signal while the cultured myoblasts were barely detectable. A dose-response curve assessed that if high numbers of SCs (500–5000 cells) were transplanted more than 80% of the mice showed engraftment, while this was only 16% if only 10 cells were transplanted. BLI also demonstrated that the cells proliferated after injection until reaching a plateau in which tissue homeostasis was obtained. Furthermore, transplanted SCs have shown to specifically proliferate in response to injury as demonstrated via ~80-fold increase in BLI signal. To establish if SCs also migrate towards an injury site after systemic injection, SCs were labeled with ^111^In-oxine [190]. First, cells were injected into uninjured rat tibialis anterior muscle and the distribution of ^111^In-labeled cells was clearly visible on SPECT from 2 to 168 h after injection. There was a continuous decrease in decay-corrected radioactivity at the transplanted site indicating diminishing cell numbers, which is in accordance with low survival rates of SCs after intramuscular injection [191]. Afterwards, labeled SCs were injected intravenous in an acute muscle injury model and healthy animals. Three hours after transplantation, barely any activity was detectable in both injured and healthy muscles. Starting from 24 h after transplantation, ^111^In-labeled cells could be retrieved in the injured leg and from 78 h until 168 h after transplantation this was significantly higher compared to healthy muscles. The therapeutic effect of the SCs was however insufficient to maintain muscle mass.

## 6. Conclusion

Stem cells have shown great potential in several neurodegenerative and neuromuscular disorders in preclinical research. Unfortunately, stem cells have not yet achieved their true potential in humans with clinical efficacy being very low or absent in all clinical studies. The number of treated patients however was too small to evaluate modest therapeutic benefit but everyone feels that there is a need for improving stem cell mediated therapy in humans. To optimize stem cell therapy, we have to understand how the cells are behaving* in vivo* and this is where imaging is of crucial importance. It allows longitudinally and noninvasively following the distribution of the cells* in vivo*.

Different cell imaging methods have been developed that even allow cell visualization on clinically used imaging devices such as PET, SPECT, and MRI. MRI provides good anatomical information but has reduced sensitivity and is mainly associated with a disconnection of the signal from its target when the labeled cells die. This results in uncertainty when interpreting the images and makes it difficult to make strong conclusions. PET and SPECT have a relatively lower spatial resolution but are highly sensitive and are readily quantifiable. Furthermore, several (human) radionuclide reporter genes have been developed. The use of reporter genes allows directly visualizing cell survival, amount, and proliferation, three factors which are crucial in evaluating the regenerative potential of stem cells. Also for the development of induced pluripotent stem cell- (iPSC-) derived therapies indirect labeling can be of great use as a safety measure to observe teratoma formation noninvasively. Furthermore, reporter genes can be placed under control of a tissue specific promoter to noninvasively monitor stem cell differentiation and functionality* in vivo*.

The major drawback of reporter gene-based imaging is the incorporation of genomic material in the cell. However, with the recent developments of site-specific genome-editing approaches, such as zinc-finger nucleases, transcription activator-like effector nucleases, and clustered regularly interspaced short palindromic repeats/caspase 9, safe incorporation of the reporter gene in dedicated areas of the genome becomes feasible which opens the doors for reporter gene-based imaging in a clinical setting [192]. Only by further exploring the possibilities of stem cell monitoring will we be able to evaluate and decipher the mode of action of stem cell therapy in different diseases.
